# Efficient conversion of solar energy to biomass and electricity

**DOI:** 10.1186/2046-9063-10-4

**Published:** 2014-06-11

**Authors:** David Parlevliet, Navid Reza Moheimani

**Affiliations:** 1 School of Engineering and Information Technology, Physics and Energy, Murdoch 6150, Western Australia, Australia; 2 Algae R&D Centre, School of Veterinary and Life Sciences, Murdoch 6150, WA, Australia; 3 Murdoch University, Murdoch 6150, WA, Australia

**Keywords:** Solar energy, Biofuel, Photovoltaics, Microalgae, Renewable

## Abstract

The Earth receives around 1000 W.m^−2^ of power from the Sun and only a fraction of this light energy is able to be converted to biomass (chemical energy) via the process of photosynthesis. Out of all photosynthetic organisms, microalgae, due to their fast growth rates and their ability to grow on non-arable land using saline water, have been identified as potential source of raw material for chemical energy production. Electrical energy can also be produced from this same solar resource via the use of photovoltaic modules. In this work we propose a novel method of combining both of these energy production processes to make full utilisation of the solar spectrum and increase the productivity of light-limited microalgae systems. These two methods of energy production would appear to compete for use of the same energy resource (sunlight) to produce either chemical or electrical energy. However, some groups of microalgae (i.e. Chlorophyta) only require the blue and red portions of the spectrum whereas photovoltaic devices can absorb strongly over the full range of visible light. This suggests that a combination of the two energy production systems would allow for a full utilization of the solar spectrum allowing both the production of chemical and electrical energy from the one facility making efficient use of available land and solar energy. In this work we propose to introduce a filter above the algae culture to modify the spectrum of light received by the algae and redirect parts of the spectrum to generate electricity. The electrical energy generated by this approach can then be directed to running ancillary systems or producing extra illumination for the growth of microalgae. We have modelled an approach whereby the productivity of light-limited microalgae systems can be improved by at least 4% through using an LED array to increase the total amount of illumination on the microalgae culture.

## Introduction

### Light

The irradiance from the sun varies widely with wavelength and has been well characterised [1]. The spectrum of the light outside the Earth’s atmosphere (the extra-terrestrial spectrum) differs from the spectrum as measured from the Earth’s surface (the terrestrial spectrum) due to absorption within the Earth’s atmosphere. There are two standard terrestrial solar spectral irradiance distributions [2] used in the testing of photovoltaic modules. These provide a standard wavelength distribution of the solar irradiance which allows the efficiency and performance of different solar modules to be measured and compared.

There are two standard spectra defined in the standards [3]. The first of these is the direct normal spectrum, which is *‘the direct component contributed to the total hemispherical (or ‘global’) radiation on a 37°-tilted surface’*[3]. The latter is a reasonable average for photovoltaics panels tilted towards the equator, in the United States of America and regions of Australia and applies to Sun facing 37°-tilted surfaces [3]. The standard defined in ASTM G-173-03 takes into account average values for the atmospheric composition, aerosols, water vapour and ozone content [2].

**Figure 1:**
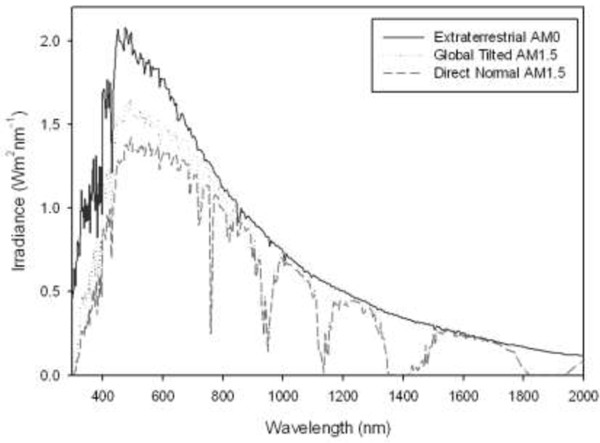
**Solar spectrum as defined in ASTM G-173-03 **[2]**.**

A plot of the extra-terrestrial irradiance and the two spectra described in the ASTM G-173-03 standard is shown in Figure 1. The irradiance is dependent on the air mass, or path length of irradiation through the atmosphere. The spectra in ASTM G-173-0 use an air mass of 1.5, which is a reasonable average for the mid-latitudes.

### Algae

Algae are plant like organisms which contain Chlorophyll *a,* have oxygenic photosynthesis and have no specialised organelles [4]. Algae divide to both prokaryotic and eukaryotic organisms and range from uni-cellular microalgae (less than 1 μm) to multicellular macroalgae (as long as 60 m) [4]. Algae can be found in any aquatic and moist habitats such as marine, freshwater and are common in soils, salt lakes and hot springs [4]. All photosynthetic algae require light to grow [5] and algal photosynthesis accounts for almost more than half of the total global primary productivity and form the basis of almost all of the aquatic food webs [6]. Microalgae have been identified as a potential source of bioenergy production [7,8]. There are many interacting factors such as light, dissolved O_2_, shear, CO_2_, pH and nutrients effecting and limiting the growth of microalgae [5]. However, light is the main limit to the growth of microalgae [9,10]. Photosynthetic pigments are responsible for absorbing light [11]. The algae are unique in the variation of these pigments in each phylum and in many classes [12]. The main reason for this diversity relates to each group ancestral lineage which has no close affinity between different groups of algae [13]. This diversity of pigments has also led to their recognition as taxonomic and phylogenetic markers [13]. Photosynthetic complexes are made up of Chlorophyll-like molecules including chlorophyll a, b, c, d and e, bacteriochlorophylls, pheophytin a and b and other kinds of pigment molecules such as carotenoid α and β, xantophylls [14]. Energy collected by the antenna complexes is transferred downward like a funnel to lower-lying, chlorophyll-containing reaction centre pigment complexes [15]. It is well known that the number of reaction centres in the photosynthetic organisms is significantly smaller than the number of pigment molecules and most of the pigments function as antenna [16]. The earth receives 3.9 × 10^6^ EJ of total solar energy each year [17]. The photosynthetic efficiency (PE) is the fraction of light energy converted to chemical energy through by photosynthetic organisms (i.e. algae, cynobacteria). The significance of PE is reliant on how light energy is defined. Actual sunlight where between photosynthetic active radiation (PAR) is only 45% and 48% of light [18], the theoretical maximum efficiency of solar energy conversion is between 11% and 12% (110 W.m^−2^ to 120 W.m^−2^). It is to be noted that, to date, the average PE is between 2% and 5% [19]. The main reason for such a difference is that a) not all light can be captured by the pigments, b) only a portion of PAR can be converted and c) light and dark respiration can reduce the overall efficiency. It is to be noted that the excess light will be discarded (i.e. as heat or fluorescence) to avoid damaging the photosynthetic apparatus.

**Figure 2:**
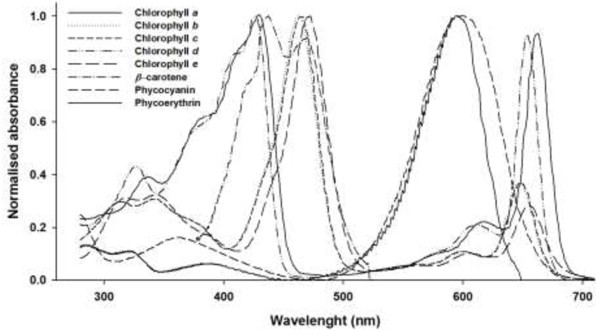
**Normalized absorbance spectra of some photosynthetic pigments **[20]**.**

The main spectral absorption peaks for Chlorophylls a, b, and c [20] and several pigments present in various microalgae are shown in Figure 2. As can be seen the absorption peaks are predominantly in the blue and red portion of the spectrum with a significant portion of the spectrum in the green range being underutilised. This portion of spectrum could be used for another purpose without impacting on the growth of the microalgae [21].

### Photovoltaics

The use of photovoltaics, or solar cells, has been described as the ‘art of converting sunlight directly into electricity’. These solid state devices take incident illumination and produce a supply of electrons which can be used by an external circuit [22]. Photovoltaic devices are produced from a range of different materials. Of these, silicon is most commonly used as a semiconductor material for producing solid state solar cells. However, there is increasing interest in other technologies, many of which have been commercialised and are in production.

The first solar cell was produced from crystalline silicon and had a conversion efficiency of 6% [23]. Through significant research and improvement, modern state of the art solar cells produced from crystalline silicon have been produced with conversion efficiencies of up to 25% and 22% in small laboratory and full modules, respectively [24,25]. In the marketplace there are several types of solar cell technologies available including crystalline, micro-crystalline and amorphous silicon. Due to their higher efficiencies and the economies of scale, the world market is dominated by crystalline silicon solar cells which held over 93.5% in 2005 [26].

### Light emitting diodes

Light emitting diodes (LEDs) are highly efficient solid state devices that can convert electrical current into light. The internal quantum efficiency of high quality LEDs can exceed 99%, however there are difficulties in extracting the light from the LED which leads to low external quantum efficiencies (EQE) in the order of a few per cent [6].

There are a range of different technologies and materials used to produce LEDs and a large amount of research going into increasing the external quantum efficiency of these LEDs. These include blue emitting InGaN-GaN LED’s with a EQE of 40% [17], thin film GaAs LEDs with a 30% EQE, [6] and organic LEDs with an EQE of 30% [27]. In some cases careful texturing can improve the light extraction efficiency which yields LEDs with an EQE greater than 50% and in some cases up to 60.9% [28].

These LEDs with high external quantum efficiencies (60.9% [28]) for particular wavelengths of light would be useful for adding additional targeted illumination to microalgae ponds.

In this work we propose introducing a filter above the algae pond to allocate the incident illumination to different purposes. The spectrum the filter transmits is allowed to be incident upon the algae pond, while the remainder is provided to a solar cell for electricity production. The nature of this *blackbox* filter and how the light is split between these two purposes is not a subject of this current work. We will assume that the filter can selectively transmit or redirect the different parts of the spectrum to different purposes with minimal energy loss. However, there are several candidate options, including a specifically tailored semitransparent thin film PV, luminescent solar concentrators, or other advanced energy harvesting flat glass panel that match the spectrum not used by the microalgae. One excellent candidate technology system can transmit arbitrary visible light wavebands, capture the infrared part of the spectrum, concentrate it on the edge of a glass panel and convert this to electricity [29].

In this work we have allocated the solar spectrum to different purposes using readily available lighting filters as model for the *blackbox* filter. The ideal spectrum to allocate to different uses will be dependent on the type of algae, response of growth rate and yield to different spectra and which parts of the spectrum are necessary for viable growth.

The models described in this work are designed to determine the portion of light that can be converted into electricity if different portions of the spectrum are redirected to the algae pond. This energy could be used to run ancillary systems in an algae pond or could be used to increase the illumination upon the pond using specific spectra generated by highly efficient LED lighting. These models also describe the potential improvement in yield if light is the limiting factor.

## Theory/Calculation

We can model the concept of transmitting a portion of the solar spectrum to a microalgae pond and converting the remainder of the light into electricity via a conventional solar panel. This allows us to determine the viability of a cultivation system based on this concept in terms of generating electivity or increasing the portion of specifically targeted PAR available for cultivation. For this model we are not concerned with how the light will be collected by a solar panel but are investigating the allocation of the solar spectrum to different purposes. To this end we will use typical commercially obtainable filters as a model for the *blackbox* filter which divides the spectrum for different purposes. What the filter transmits will be allocated to the algae, what the filter does not transmit will be allocated to the solar cell. The ideal spectrum to allocate to different uses will be dependent on the type of algae, response of growth rate and yield to different spectra and which parts of the spectrum are necessary for viable growth and will be tailored in future experimental work.

**Figure 3:**
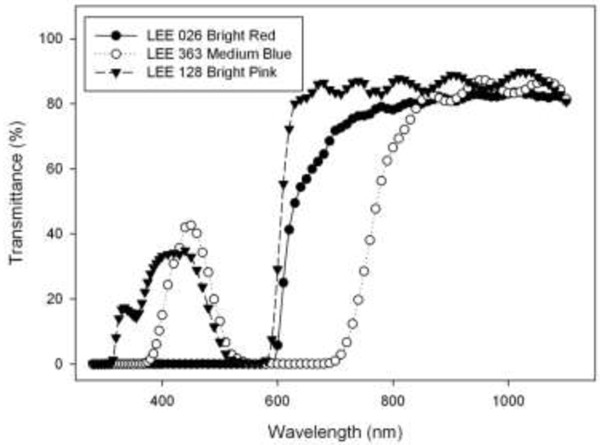
Transmittance of filters as measured with ultraviolet and visible spectroscopy.

The filters used in this study are LEE 026 Bright Red, LEE 363 Medium Blue and LEE 128 Bright Pink filters. The transmission of each of these was measured using ultraviolet and visible spectroscopy and is shown below in Figure 3.

For photovoltaic devices, the absorption of light is determined by the band-gap. This needs to be close to the peak of the energy range of the AM1.5 spectrum (1 eV to 3 eV) and not all semiconductors are suitable for use as solar cells, the most suitable band-gap for which is about 1 to 1.6 eV [22]. Although photovoltaic devices would work with a higher efficiency if they only had to absorb monochromatic light [30], normally photovoltaic devices are designed to absorb as much of the solar spectrum as possible. The response to different components of the solar spectrum can be measured using the spectral response technique.

The spectral response of a solar cell is usually defined as the output current under short-circuit conditions per unit power of incident monochromatic light as a function of wavelength [31] and is a measure of the proportion of charge carriers generated by the incident photons.

**Figure 4:**
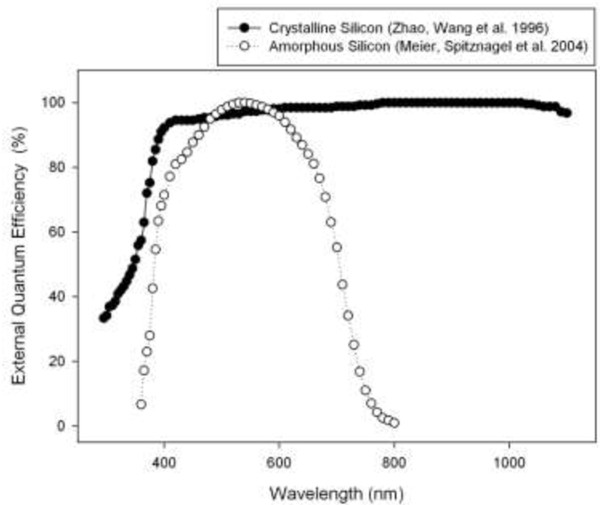
The external quantum efficiency of crystalline and amorphous silicon solar cells.

There are several different technologies used in photovoltaic devices, including but not limited to: crystalline silicon [24,25], amorphous silicon [32,33] and microcrystalline silicon [34,35]. The external quantum efficiency curves of current state of the art single crystal silicon solar cells with an efficiency of 24% [36] and a highly efficient single junction amorphous silicon device with a stable efficiency of 9.47% [33] are shown in Figure 4.

From the reported spectral response graphs and parameters for crystalline silicon [25] and amorphous silicon [33] we can calculate the power generated if the light not transmitted by the filters is allowed to fall on the solar cell.

The short circuit current density (J_SC_) generated by the solar cell is calculated from:

$$J_{\mathrm{SC}}=\int \mathrm{EQE}\left(\lambda \right)\left(\frac{\Phi {\left(\lambda \right)}_{\mathrm{AM}1.5}\left(T\left(\lambda \right)\right)}{q}\right)$$

Where EQE(λ) is the external quantum efficiency as a function of wavelength, Φ(*λ*)_*AM*1.5_ is the photon flux density calculate from the AM1.5 (Global Tilted) solar spectrum, *T*(*λ*) is the transmittance of the filter as measured from spectroscopy measurements and *q* is the charge of an electron.

The open circuit voltage (V_OC_) of the solar cell is also dependent on the short circuit current density and will vary with the irradiance incident upon the cell. This can be calculated from [37]:

$$V_{\mathrm{OC}}=\frac{\mathrm{kT}}{q}\ln\left(\frac{J_{\mathrm{SC}}+J_{0}}{J_{0}}\right)$$

Where E_g_ is the band-gap of the semiconductor material, k is Boltzmann’s constant, T is the cell temperature in K and J_0_ is the inferred from the published parameters of each device.

The power generated (P) in W.m^−2^ from the cell is then:

$$P=\mathrm{FF}.l_{\mathrm{SC}}.V_{\mathrm{OC}}$$

Where the fill factor (*FF*) is the value published in the literature for each cell type.

The power generated by the solar cell could either be used to run ancillary systems or directed into additional lighting. To ensure that the additional lighting is appropriate for the needs of the microalgae Light Emitting Diode (LED) arrays can be used.

The additional PAR power (P_PAR_) in Wm^−2^ that can be produced using the power generated (P_in_) using the system modelled above can be calculated from:

$$P_{\mathrm{PAR}}=\mathrm{EQ}E_{\mathrm{LED}}.P_{\mathrm{in}}$$

Where EQE_LED_ is the external quantum efficiency of the LED.

## Results

**Figure 5:**
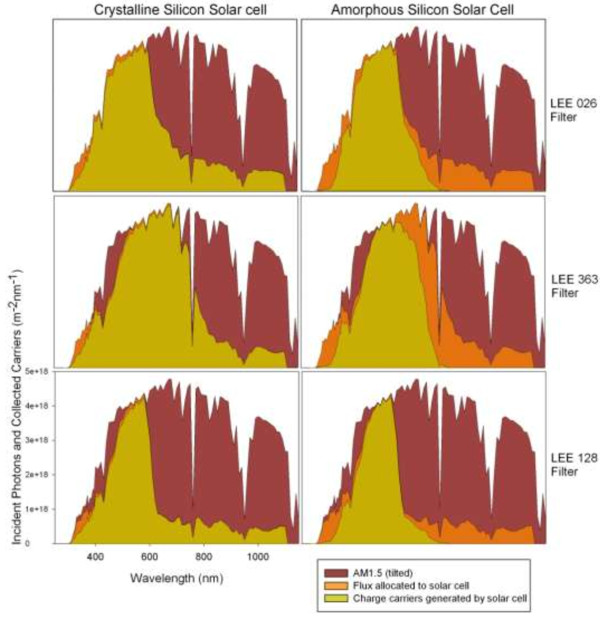
Photon flux and carriers collected for each of the solar cell technologies and filters used.

The spectrum of light passing through three different filters has been modelled as outlined above. The number of carriers generated by each of the solar cell technologies (amorphous silicon and crystalline silicon) when allocated the spectra not transmitted by the *blackbox* filter are shown in Figure 5.

The power from the spectra transmitted through the various filters and onto the algae is shown below in Table 1. If no filter was placed on top of the algae, 431 W.m^−2^ or 1982 μ mole photons.s^−1^.m^−2^ would be available for use in the PAR section of the solar spectrum. The calculations from the three filters reflect the fact that smaller portions of the solar spectrum are being transmitted to the algae. Under AM1.5 illumination the highly efficient crystalline silicon cell would produce more electrical power than a less efficient amorphous silicon solar cell. However, from Figure 5 and Table 1 it can be seen that in some cases the amorphous silicon cell will generate more electrical output. This is due to the difference in spectral response between the two technologies. It has been previously shown that amorphous silicon solar cells are more suitable for “blue” spectra while crystalline cells are more suitable for “red” spectra [38]. The higher output using the LEE128 Bright Pink filter can be explained as the filter absorbs a less of the red end of the spectrum than the other filters, which is the region where the amorphous silicon cell has a lower external quantum efficiency than the crystalline silicon device.

**Table 1:** PAR power transmitted to algae and electrical power generated by solar cells

**Filter**	**PAR power transmitted to algae (W.m**^**−2**^**)**	**Power generated by crystalline silicon (W.m**^**−2**^**)**	**Power generated by amorphous silicon (W.m**^**−2**^**)**
**None**	431.03	0	0
**LEE 026 Bright red**	71.8	121.13	81.54
**LEE 363 Special medium blue**	47.08	151.52	93.46
**LEE 128 Bright pink**	147.97	48.93	64
**Black (absorbs all light)**	0	239.85	94.69

As shown in Table 2, if the electricity is used to produce additional light to illuminate the algae the total amount of PAR power can be increased when compared to the PAR transmitted through the filter. The figures shown in Table 2 are from the power generated by a crystalline silicon solar cell as they produce a larger amount of electrical power in most situations. The values for PAR in μ mole photons.s^−1^.m^−2^ have also been calculated and are shown in Table 3. For the calculation of the additional PAR generated by the LED a wavelength of 650 nm has been used. This is in the range of what can be absorbed efficiently be algae and can be generated efficiently by LEDs. It should be noted that this value decreases if LEDs are used that emit a shorter wavelength radiation. The value in W.m^−2^ (Table 2) is independent of the wavelength of the LED and is used in this work to remove the ambiguity associated with wavelength.

**Table 2:** PAR power transmitted to algae and electrical power generated by crystalline silicon solar cells

**Filter**	**PAR power transmitted to algae (W.m**^**−2**^**)**	**Power generated by crystalline silicon (W.m**^**−2**^**)**	**Additional PAR power generated by LED (W.m**^**−2**^**)**	**Total PAR power (W.m**^**−2**^**)**
**None**	431.03	0	0	431.03
**LEE 026 Bright red**	71.8	121.13	72.68	144.48
**LEE 363 Special medium blue**	47.08	151.52	90.92	138
**LEE 128 Bright pink**	147.97	48.93	29.36	177
**Black (absorbs all light)**	0	239.85	143.92	143.92

The additional PAR power produced by an LED with an EQE of 60% has also been calculated. The Total PAR Power is the sum of the PAR transmitted to the algae and the PAR generated by the LEDs.

**Table 3:** PAR transmitted to algae and electrical power generated by crystalline silicon solar cells

**Filter**	**PAR transmitted to algae (μ mole photons.s**^**−1**^**.m**^**−2**^**)**	**Power generated by crystalline silicon (W.m**^**−2**^**)**	**Additional PAR generated by LED (μ mole photons.s**^**−1**^**.m**^**−2**^**)**	**Total PAR (μ mole photons.s**^**−1**^**.m**^**−2**^**)**
**None**	1982.13	0	0	1982.13
**LEE 026 Bright red**	393.71	121.13	394.66	788.38
**LEE 363 Special medium blue**	179.26	151.52	493.68	672.94
**LEE 128 Bright pink**	738.34	48.93	159.43	897.78
**Black (absorbs all light)**	0	239.85	781.48	781.48

The additional PAR produced by an LED with an EQE of 60% at 650 nm has also been calculated.

## Discussion

There is no doubt that the current fossil fuel resources are depleting. Sustainable alternative energy can replace some of our increasing energy need. One way to meet the need for an alternative, renewable liquid fuel is the production of bioethanol and biodiesel. Microaglae have been suggested as a raw material for bioenergy production. In general, sun light is the main source of all available energy on earth. Light is also the main element for the process of photosynthesis and is the main limiting factor for microalgae growth. Photosynthesis uses approximately 25% of the solar spectrum (mostly blue and red wavelengths); the rest is either reflected or heats up the growth media. The use of photovoltaics, or solar cells, is a second well established method for converting light into useable energy and has been described as the ‘art of converting sunlight directly into electricity’ [22]. Whereby, a highly efficient crystalline silicon solar cell can convert up to 24% of the solar spectrum into electricity [36].

These two methods of energy production would appear to compete for use of the same energy resource (sunlight) to produce either chemical or electrical energy. However, some groups of microalgae (i.e. Chlorophyta) generally only require the blue and red portions of the spectrum whereas solar cells are generally produced with a very broad spectral response to be able to efficiently collect as much of the solar spectrum as possible. Some technologies have a broader response than others as illustrated in Figure 4. By using the AM1.5 direct solar spectrum as a baseline we can model the amount of energy in the incident irradiation that could be converted into electricity by a hypothetical lossless system that diverts part of the solar spectrum to an algae pond and part to a solar cell. For this model we have used readily available lightning filters as proxies for light splitting devices. The spectra the filter transmitted was provided to the algae while the portion absorbed by the filter was allocated to the solar cell.

The addition of a *blackbox* filter above an algae pond has been modelled and it has been shown in Table 1 that introducing this filter reduces the total amount of PAR power that is incident upon the algae pond. Without this filter in place a PAR power of 431 W.m^−2^ is available for algae growth. With the filter in place the available PAR power is reduced to between 47 W.m^−2^ and 148 W.m^−2^.

However, not all PAR is utilised by the algae, as it does not correspond with the absorption peaks of the chlorophylls and pigments as shown in Figure 2. From Figure 2 it can be seen that only a portion of the PAR spectrum is actually absorbed by the chlorophylls and pigments common in microalgae. If an alga such as Chlorella with Chlorophylls a and b and β-carotene, is used it is a reasonable approximation that only 170 W.m^−2^ is required for this process, the remainder is not fully utilised. This can be calculated under the assumption that the largest contribution to photosynthesis is from regions where the normalised absorbance is greater than 60% and the remainder is not critical for growth. For example, the normalised absorption of Chlorella with Chlorophylls a and b and β-carotene, is greater than 60% in the regions of 378 – 485 nm and 654 – 670 nm. The amount of solar energy contained in these regions of the AM1.5 spectrum (see Figure 1) is 170 W.m^−2^. Thus, a reduction in the amount of PAR may not necessarily effect the growth of the algae, provided the reduction is in the parts of the spectrum the algae do not require for growth or photosynthesis. This is an area that requires further experimental work to determine which parts of the spectrum are needed by the algae and which can be allocated to electricity production.

Based on the model described, using a LEE Special Medium Blue *blackbox* filter, up to 151 W.m^−2^ is generated using a highly efficient crystalline silicon solar cell. This can be allocated to running ancillary systems such as pumps, or directed back into an LED array to provide additional illumination to the algae culture. LEDs have been reported with external quantum efficiencies up to 60% [28] in the lab. If the power generated by the solar cells is used to generate additional illumination by these LEDs in the parts of the spectrum the algae require for growth the amount of PAR available to the algae can be increased. For the systems modelled above, this increases the total amount of PAR available to the algae up to 177 W.m^−2^. Thus, the total amount of useable PAR incident on an algae pond can be increased by introducing a filter system above an algae pond that directs part of the light to the algae and part to a highly efficient crystalline silicon solar cell and using the generated electricity to produce additional targeted PAR.

The amount of energy production has been calculated for the filter and solar cell combination modelled above that generates the largest amount of total PAR power. These calculations indicate up to 49 Wm^−2^ under AM1.5 (global tilted) radiation or 241 to 482 MJm^−2^y^−1^, depending on location in Australia, can be generated by a highly efficient crystalline silicon solar cell which when coupled with an LED array and the light passing through the filter allows 177 W.m^−2^ of PAR to illuminate the algae. Assuming a photosynthetic biofuel production rate of a green alga (i.e. *Chlorella*) with Chlorophylls *a* and *b* and β-carotene = 20 g.m^−2^.d^−1^[7], which is equal to around 2-6% photosynthetic efficiency [39], and a calorific energy content of 15–20 MJkg^−1^, the annual production rate of biofuel will be between 109 and 146 MJm^−2^ year^−1^. In a light limited system, by increasing the amount of useable PAR, this could be increased. If only 170 W.m^−2^ is required for achieving a successful productivity of 20 g.m^−2^.d^−1^ the increased illumination (177 W.m^−2^) modelled here could potentially increase the annual algae productivity rate by at least 4%. This may be improved if the selection of microalgae is such that the biofuel production of 20 g.m^−2^.d^−1^ can be achieved using just the portion of the spectrum absorbed by Chlorophyll *a.*

There are a number of assumptions and limitations in this model. For the total amount of useable PAR with the proposed filter system to be increased relative to the useable PAR without the filter we require a few high efficiency devices. Firstly, the solar cells need to be able to absorb the portions of the spectrum provided to them with very high quantum efficiency and preferably have a broad wavelength response. This would increase the absorption of light in the blue and red portions of the spectrum which are outside of the range of PAR (400 nm - 700 nm). The modelled system also relies on highly efficient LEDs. These can be produced in the lab with external quantum efficiencies ranging up to 60% [28]. A LED with a higher efficiency will be able to generate larger amounts of useable PAR which would improve the growth of the algae. The filter that is used to allocate the solar spectrum to different uses needs to be tailored to the species of algae being cultivated. By doing so, larger portions of the solar spectrum can be directed to the solar cells and thus generating more useable PAR via the LEDs.

We recognise that, if the solar cells are in the order of 25% efficient, they would be producing more electrical energy per year from the spectrum they receive than the algae are storing as energy. However, the advantage of our proposed method is the production of chemical energy for transportation. We also recognise that the power used to produce extra illumination could be provided by a connection to the electricity grid. However, microalgae are often cultivated in remote areas where an independent power supply may be advantageous. On the other hand, some cultivation systems may have limited amounts of land available for cultivation. In this situation, co-locating the solar array and the algae ponds would result in an increased growth. Additionally there are some other benefits from filtering the light before it is incident upon the algae. The removal of the parts of the spectrum not required by the algae would reduce the heating and evaporation from the algae pond.

## Conclusions

Whilst other factors can influence actual productivity, photosynthesis stipulates the potential upper limit on the effectiveness with which solar energy can be transformed into stored chemical energy (i.e. biofuel). As such, the solar spectrum is the ultimate source of all biomass production. Solar panels have also been recognised as a potential electrical energy production system. By combining two energy production systems we can fully utilize the solar spectrum and light incident on a surface. This would allow both the production of biofuel and electricity from the one facility making efficient use of available land. We propose introducing a filter above the algae pond that will allocate the incident illumination to different purposes. We have modelled this by using commercially available lighting filters as *blackbox* filters. This hypothetical filter allows the transmitted light through to the algae and used the remainder to generate electricity using either a highly efficient crystalline silicon solar cell or an amorphous silicon solar cell. Using this system up to 151 W.m^−2^ of electrical power can be produced. Introducing a method of cogeneration of electrical energy has benefits in the often remote areas microalgae cultivation occurs. This can reduce the costs associated with production, dewatering and extraction of oil from microalgae. This allows for the cheaper and more efficient production of biofuel or value added crops in remote locations which are located away from sources of electrical power. Additionally, we have shown that the total amount of useable PAR incident on an algae pond can be increased by introducing a filter system above an algae pond that directs part of the light to the algae and part to a highly efficient crystalline silicon solar cell and using the generated electricity to produce additional targeted PAR. This can result in a total available PAR of 177 W.m^−2^ which can be used to increase the productivity and value of algae cultivation.

## Competing interests

The authors declare that they have no competing interests.

## Authors’ contributions

DP and NM jointly developed the model and conceptual framework for the distribution of solar radiation between microalgae and photovoltaics. DP performed numerical modelling and drafted the manuscript. NM contributed specifically in the area of biology and photosynthesis. Both authors read and approved the final manuscript.
